# Affibody-Drug Conjugates Targeting the Human Epidermal Growth Factor Receptor-3 Demonstrate Therapeutic Efficacy in Mice Bearing Low Expressing Xenografts

**DOI:** 10.1021/acsptsci.4c00402

**Published:** 2024-09-12

**Authors:** Jie Zhang, Sara S. Rinne, Wen Yin, Charles Dahlsson Leitao, Elvira Björklund, Ayman Abouzayed, Stefan Ståhl, John Löfblom, Anna Orlova, Torbjörn Gräslund, Anzhelika Vorobyeva

**Affiliations:** † Department of Protein Science, KTH Royal Institute of Technology, Roslagstullsbacken 21, 114 17 Stockholm, Sweden; ‡ Department of Medicinal Chemistry, Uppsala University, Dag Hammarskjöldsv 14C, 751 83 Uppsala, Sweden; § Science for Life Laboratory, Dag Hammarskjöldsv 14C, 751 83 Uppsala, Sweden; ∥ Department of Immunology, Genetics and Pathology, Uppsala University, Dag Hammarskjölds Väg 20, 751 85 Uppsala, Sweden

**Keywords:** affibody molecule, HER3, DM1, affibody-drug conjugate, biodistribution

## Abstract

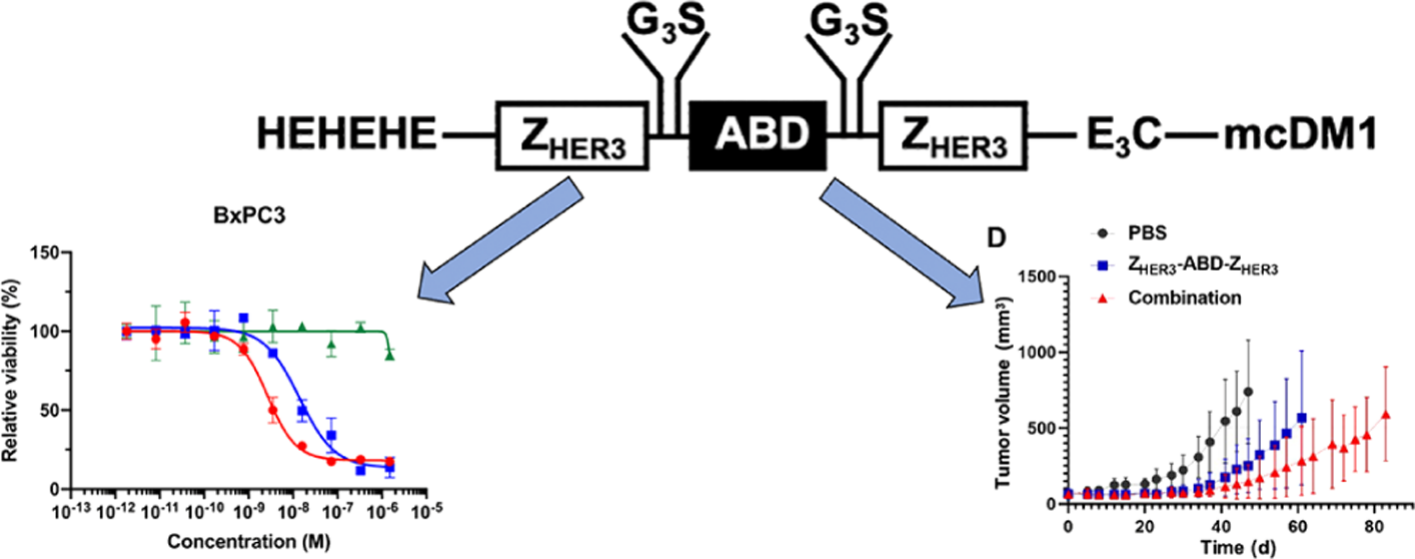

The outcome of clinical trials evaluating drugs targeting the human epidermal growth factor receptor 3 (HER3) has been poor, with primary concerns related to lack of efficacy. HER3 is considered a difficult target since its overexpression on tumors is relatively low and there is normal expression in many different organs. However, a significant number of patients across different cancer indications have overexpression of HER3 and the development of novel modalities targeting HER3 is therefore warranted. Here, we have investigated the properties of affibody-based drug conjugates targeting HER3. The HER3-targeting affibody molecule Z_HER3_ was fused in a mono- and bivalent format to an engineered albumin-binding domain (ABD) for *in vivo* half-life extension and was coupled to the cytotoxic drug DM1 *via* a non-cleavable maleimidocaproyl (mc) linker. *In vivo*, a moderate uptake was observed for [^99m^Tc]Tc-labeled Z_HER3_-ABD-Z_HER3_-mcDM1 in HER3 expressing BxPC3 tumors (3.5 ± 0.3%IA/g) at 24 h after injection, and clearance was predominately renal-mediated. Treatment of mice with BxPC3 human pancreatic cancer xenografts showed that a combination of Z_HER3_-ABD-Z_HER3_-mcDM1 and its cytostatic analog Z_HER3_-ABD-Z_HER3_ was efficacious and superior to treatment with only Z_HER3_-ABD-Z_HER3_, providing tumor growth inhibition and longer median survival (90 d) in comparison to monotherapy (68 d) and vehicle control (49 d). Z_HER3_-ABD-Z_HER3_-mcDM1 was found to be a potent drug conjugate for the treatment of HER3-expressing tumors in mice.

Cytotoxic drugs are widely used in cancer therapy for killing rapidly dividing cancer cells. Since these drugs are given systemically, highly proliferating normal cells, *e.g.*, in the bone marrow, might be affected as well, resulting in adverse events and a narrow therapeutic window. One approach to prevent the cytotoxicity on normal cells is to attach the drugs to affinity proteins, specifically targeting overexpressed surface receptors on the cancer cells, to allow tumor-specific delivery. The most well-studied format is the antibody-drug conjugate (ADC), where a monoclonal antibody (mAb) is used as the targeting agent for the cytotoxic drugs.^1^ In recent years, antibody fragments, engineered scaffold proteins (ESPs), and peptides have also shown promise as carriers of cytotoxic drugs.^2,3^

The human epidermal growth factor receptor (HER) family, includes the receptor tyrosine kinases HER1/EGFR, HER2/ErbB2, HER3/ErbB3, and HER4/ErbB4. They are activated upon the formation of homo- or heterodimers with other family members, followed by cross-phosphorylation of their intracellular domains. HER3 has two main activating ligands, heregulin (HRG) and neuregulin 2 (NGR2). The kinase activity of HER3 is weak but it can form potent signaling complexes when heterodimerized with other members of the HER family. One of the most tumorigenic heterodimers is formed between HER2 and HER3, which strongly activates the PI-3K/Akt pathway, a well-known signaling pathway driving tumorigenesis.^4^ The members of the HER family might be abnormally active in different cancers, sometimes as a consequence of upregulated expression, and may then serve as targets for tumor-specific delivery of cytotoxic drugs for these specific subsets of patients.

HER2 is a target for ADCs, with trastuzumab emtansine (T-DM1) available for clinical use in patients with HER2 overexpressing breast cancer, and trastuzumab deruxtecan for patients with HER2 expressing breast cancer, gastric cancer, and gastroesophageal adenocarcinoma.^5^ HER3, however, has proven to be a more difficult target. It is typically overexpressed at a relatively low level, up to 50,000 receptors/cell,^6^ compared with HER2 (2 million receptors/cell).^7^ As of today, there are no HER3-targeting drugs approved for clinical use. HER3-targeting therapy has been evaluated in a clinical phase II trial with the monoclonal antibody (mAb) seribantumab in combination with paclitaxel for the treatment of ovarian cancer. However, the trial failed due to poor efficacy, where the endpoints were not met.^8^ An ADC targeting HER3, patritumab deruxtecan (HER3-DXd), is currently under clinical evaluation, in patients with HER3-expressing metastatic breast cancer.^9^

The drugs included in ADCs and other types of targeted drug conjugates are more cytotoxic than classical chemotherapeutic agents. The maytansine-derivative DM1 was initially investigated as a nontargeted chemotherapeutic drug but was found to give rise to unacceptable side effects.^10^ DM1 inhibits tubulin polymerization and is, therefore, toxic to all cells but potentially more toxic to rapidly dividing cells since it can prevent the mitotic spindle from forming during cell division. In T-DM1, the DM1 payload is conjugated *via* a non-cleavable linker. Upon engaging HER2, T-DM1 is internalized and transported to the lysosome for degradation and drug release, thereby increasing the therapeutic index and minimizing normal tissue exposure compared to DM1 alone.^5^

Due to the unmet clinical need of HER3 targeted therapies, we have in this study investigated the influence of molecular design on a HER3-targeting drug conjugate based on an engineered scaffold protein (ESP) carrying a DM1 payload. ESPs are usually small, easily engineered, and may often be produced in simple host organisms, such as *Escherichia coli*, at a low cost.^11^ In addition, many of the scaffolds used to derive ESPs, are thermostable and have low immunogenicity.^12,13^ As carriers of cytotoxic drugs, several ESPs, including affibody molecules, ADAPTs, and DARPins, have undergone preclinical evaluation, with potent responses recorded in different murine models.^14−16^ Although not an archetypical ESP, the Bicycle affinity proteins, consisting of a peptide attached to an organochemical scaffold structure, have entered clinical evaluations as drug conjugates with promising results.^17^ Compared to mAbs, the ESPs are markedly smaller and should extravasate and penetrate solid tumors more efficiently.^18^ However, due to their small size, ESPs are often rapidly excreted through kidney filtration and, therefore, have a short *in vivo* half-life. For therapeutic applications, an extended *in vivo* half-life is usually desirable since it may increase the bioavailability and, thereby, the therapeutic effect of the drug molecule. One strategy to prolong the *in vivo* half-life of small protein domains is to attach an albumin-binding domain (ABD).^19^ A commonly used ABD is the engineered ABD_035_ with a molecular weight of 5 kDa and a strong affinity to serum albumin.^20^ ABD binds to serum albumin in blood to form a complex larger than the cutoff of the glomerular filter in the kidneys, and filtration is thereby significantly reduced. Furthermore, albumin is rescued from lysosomal degradation by cells in contact with blood by utilizing pH-dependent binding to the neonatal Fc receptor (FcRn). Albumin-binding molecules, including an ABD, will similarly be rescued by FcRn, which further extends their *in vivo* half-life.

Affibody molecules are ESPs consisting of 58 amino acids arranged in a three-helix bundle structure with a molecular weight of 6.5 kDa. Previously, the affibody molecule Z_HER3:08698_, binding specifically to HER3 with 21 pM affinity, was isolated by cell sorting from a large combinatorial affibody library displayed on staphylococci.^21^ The properties of Z_HER3:08698_ have been evaluated *in vitro*, where it was found to compete with the natural HER3-ligand HRG.^22^ The targeting properties, biodistribution, and cytostatic effect of several different formats of ABD-fused HER3-targeting affibody molecules have previously been investigated *in vivo* using xenografted HER3-expressing BxPC3 tumors.^23−26^ The therapeutic efficacy of the monovalent Z_HER3:08698_-ABD and the bivalent Z_HER3:08698_-ABD-Z_HER3:08698_ was shown to be comparable to the mAb seribantumab.^25,26^ To further increase the potency of the HER3-targeting affibody constructs, we have previously constructed and investigated the characteristics of an affibody-drug conjugate, Z_HER3:08698_-ABD-mcDM1, consisting of Z_HER3:08698_-ABD functionalized with a DM1 payload.^27^ Since it was previously shown that the internalization rate of Z_HER3:08698_-ABD-Z_HER3:08698_ is more rapid than the internalization rate of Z_HER3:08698_-ABD,^23^ we hypothesized that Z_HER3:08698_-ABD-Z_HER3:08698_ may have a stronger cytotoxic effect when functionalized with DM1 compared to Z_HER3:08698_-ABD.

In this study, we have compared the cytotoxic potential of a monovalent and a bivalent HER3-targeting affibody drug conjugate, on the HER3-expressing cell lines BxPC3 and DU145. Similar to our earlier studies, a unique cysteine was introduced at the C-terminus of the fusion proteins, Z_HER3:08698_-ABD, and Z_HER3:08698_-ABD-Z_HER3:08698_, for site-specific attachment of DM1. To evaluate the rate of cell internalization, the biodistribution, and tumor targeting properties, a tag with the amino acid sequence His-Glu-His-Glu-His-Glu was placed at the N-terminus of both fusion proteins to enable chelation of [^99m^Tc]Tc. After extensive characterization of the properties of the drug conjugates *in vitro* and *in vivo*, the therapeutic potential of the bivalent construct was evaluated by treatment of mice carrying BxPC3-derived tumors.

## Results

### Production and Biochemical Characterization of the Constructs

**Figure 1:**
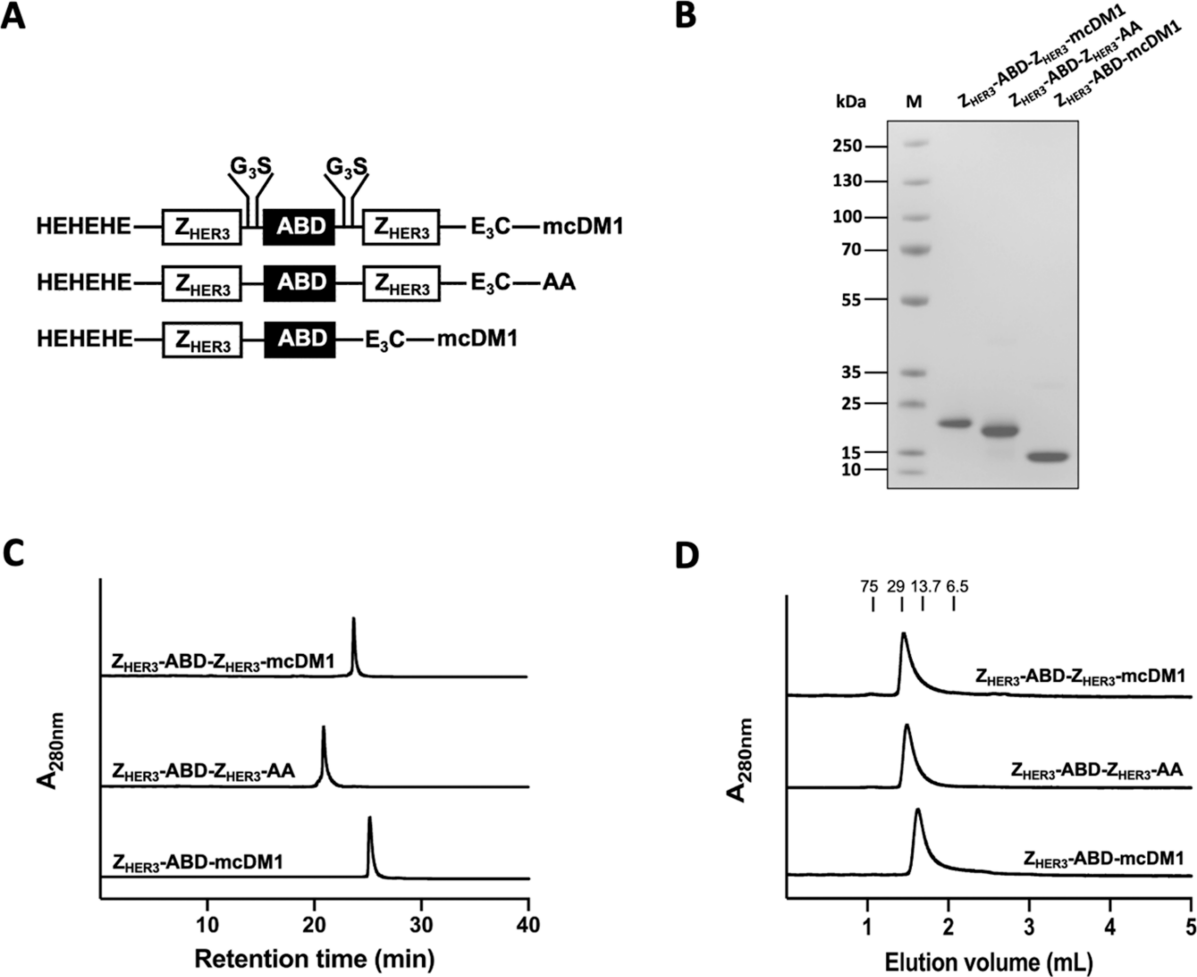
Schematic representation and biochemical characterization of the anti-HER3 affibody constructs. (A) The composition of the three constructs, Z_HER3_-ABD-Z_HER3_-mcDM1, Z_HER3_-ABD-Z_HER3_-AA, and Z_HER3_-ABD-mcDM1, from top to bottom. Each construct consists of one or two affibody molecules and an ABD connected by G_3_S linkers (only shown for the top construct). In each construct, a HEHEHE-tag is in the N-terminus for the chelation of [^99m^Tc]Tc, and an E_3_C-tag (amino acid sequence EEEC) is situated in the C-terminus for conjugation of DM1 *via* an mc-linker. (B) A picture of an SDS-PAGE gel, where lane M shows the separation of marker proteins with molecular weights indicated to the left. (C) Analysis of the constructs by RP-HPLC. The proteins were eluted using a 20–60% gradient of acetonitrile in water over 40 min. (D) Analytical size-exclusion chromatography analysis of the conjugates with PBS as running buffer. The numbers above the chromatograms indicate the molecular weights of reference proteins.

The HER3-binding and ABD-fused affibody constructs, Z_HER3_-ABD-Z_HER3_-E_3_C and Z_HER3_-ABD-E_3_C, were produced in *E. coli* and purified by affinity chromatography with HSA (human serum albumin) as immobilized ligand. The cytotoxic payload, mcDM1, was conjugated to the unique C-terminal cysteine residue of both fusion proteins, yielding Z_HER3_-ABD-Z_HER3_-mcDM1 and Z_HER3_-ABD-mcDM1. A nontoxic control, Z_HER3_-ABD-Z_HER3_-AA, was generated by the alkylation of the C-terminal cysteine of Z_HER3_-ABD-Z_HER3_-E_3_C (AA, in the name, thus represents alkylation). The composition of the affibody constructs is shown in Figure 1A.

The constructs were analyzed by SDS-PAGE to investigate the purity. As shown in Figure 1B, the constructs migrated at the expected molecular weights and showed a high level of purity. The purity of the three constructs was further investigated by reversed-phase high-performance liquid chromatography (RP-HPLC) and was found to be more than 95% pure by quantification of the area-under-curve (AUC) in the chromatograms (Figure 1C). In Figure 1C, Z_HER3_-ABD-Z_HER3_-mcDM1 was eluted later than Z_HER3_-ABD-Z_HER3_-AA, showing an increase in hydrophobicity, which is likely the consequence of the conjugation with DM1. Furthermore, Z_HER3_-ABD-mcDM1 was eluted later than Z_HER3_-ABD-Z_HER3_-mcDM1, showing that the increase in hydrophobicity imposed by DM1, is more pronounced for the smaller drug conjugate. Size-exclusion chromatography analysis was performed to investigate the mono/multimeric state of the constructs. The chromatograms showed relatively symmetrical peaks, eluted with the expected retention of a monomer, suggesting that the constructs were essentially in a monomeric state (Figure 1D). Moreover, the molecular weights of the constructs were determined by liquid chromatography–mass spectrometry (LC-MS), and showed peaks of the correct molecular weights (Figure S1). The LC-MS analysis also revealed that the N-terminal methionine had been removed from Z_HER3_-ABD-Z_HER3_-E_3_C but not from Z_HER3_-ABD-E_3_C during *E. coli* production.

### Evaluation of the Affinity to HER3 and Murine ErbB3

The affinity of the constructs to the extracellular domains of HER3 and murine ErbB3 (mErbB3) was investigated by surface plasmon resonance (SPR) binding analysis. The acquired sensorgrams are presented in Figure S2, and the derived kinetic constants from the analysis of the sensorgrams are displayed in Table 1. The affinities of Z_HER3_-ABD-Z_HER3_-mcDM1, Z_HER3_-ABD-Z_HER3_-AA, and Z_HER3_-ABD-mcDM1 to HER3 were similar, with equilibrium dissociation constants (*K*_D_ values) of 3 to 4 nM. Due to more rapid dissociation, the affinity of Z_HER3_-ABD-Z_HER3_-mcDM1 and Z_HER3_-ABD-mcDM1 to mErbB3 was up to ten times weaker than the affinity to HER3, and four times weaker in the case of Z_HER3_-ABD-Z_HER3_-AA.

**Table 1:** Kinetic Constants of the Interactions between the Anti-HER3 Drug Conjugates and HER3 and mErbB3, Derived from the Sensorgrams in Figure S2

analyte	ligand	*k*_a_ (1/Ms)	*k*_d_ (1/s)	*K*_D_ (M)
HER3	Z_HER3_-ABD-Z_HER3_-mcDM1	4.6 × 10^4^	1.5 × 10^–4^	3.2 × 10^–9^
	Z_HER3_-ABD-Z_HER3_-AA	6.3 × 10^4^	1.7 × 10^–4^	2.7 × 10^–9^
	Z_HER3_-ABD-mcDM1	4.0 × 10^4^	1.6 × 10^–4^	4.0 × 10^–9^
mErbB3	Z_HER3_-ABD-Z_HER3_-mcDM1	1.5 × 10^4^	3.8 × 10^–4^	2.5 × 10^–8^
	Z_HER3_-ABD-Z_HER3_-AA	3.2 × 10^4^	3.8 × 10^–4^	1.2 × 10^–8^
	Z_HER3_-ABD-mcDM1	1.0 × 10^4^	3.8 × 10^–4^	3.8 × 10^–8^

### *In Vitro* Cytotoxicity

**Figure 2:**
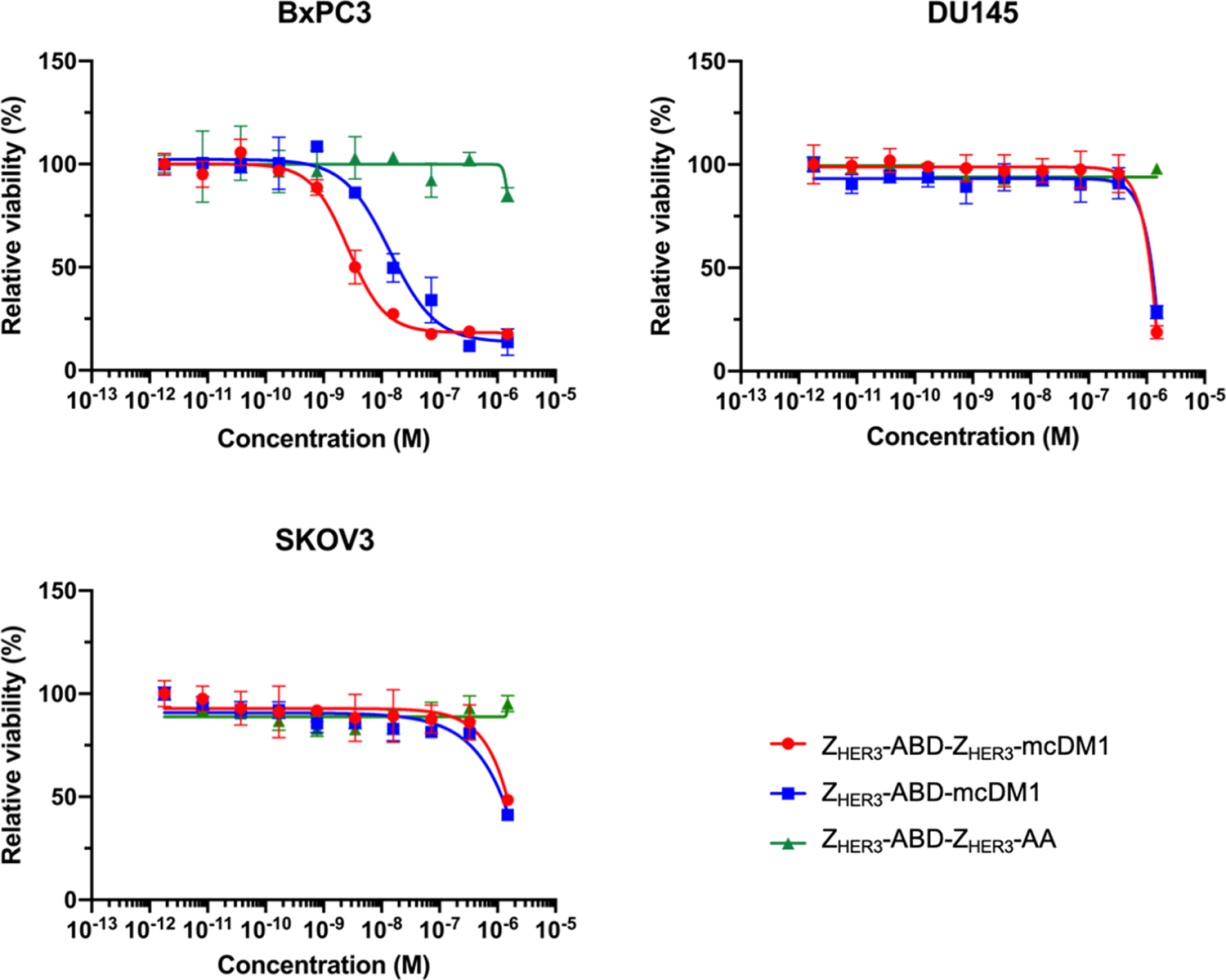
Determination of the cytotoxic effect. The cell lines indicated over each panel were incubated with dilution series (1500, 330, 73, 16, 4 nM, 800, 170, 37, 8, and 2 pM) of the constructs, Z_HER3_-ABD-Z_HER3_-mcDM1, Z_HER3_-ABD-mcDM1, or Z_HER3_-ABD-Z_HER3_-AA for 72 h. The viabilities were then measured and displayed as a fraction of the viability of cells incubated without any construct, which was set to 100%. Each data point corresponds to the average of four individual experiments.

The cytotoxic action of the anti-HER3 drug conjugates on BxPC3 (medium/low HER3 expression, 12,000 receptors per cell^28^), DU145 (low HER3 expression), and SKOV3 (HER3-very low) cells was determined, and the results are displayed in Figure 2.

Z_HER3_-ABD-Z_HER3_-mcDM1 and Z_HER3_-ABD-mcDM1 showed a dose-dependent cytotoxic effect on the BxPC3 cell line, with Z_HER3_-ABD-Z_HER3_-mcDM1 being the most cytotoxic. Z_HER3_-ABD-Z_HER3_-mcDM1 had an IC_50_ value of 3 nM, and Z_HER3_-ABD-mcDM1 had an IC_50_ value of 14 nM. For DU145 and SKOV3 cells, a cytotoxic effect was found only for the highest concentration (1500 nM) for Z_HER3_-ABD-Z_HER3_-mcDM1 and Z_HER3_-ABD-mcDM1. No cytotoxic effect was detected for the nontoxic control, Z_HER3_-ABD-Z_HER3_-AA, on any of the cell lines.

### Labeling with [^99m^Tc]Tc and Determination of Label Stability

Encouraged by the potent cytotoxic effect of the bivalent Z_HER3_-ABD-Z_HER3_-mcDM1, it was investigated further. To be able to characterize its interaction with cells *in vitro* and *in vivo*, it was radiolabeled with [^99m^Tc]Tc. The nontoxic Z_HER3_-ABD-Z_HER3_-AA was included as a control. The yield after radiolabeling was determined by ITLC and was found to be 60 ± 20% for [^99m^Tc]Tc-Z_HER3_-ABD-Z_HER3_-mcDM1 and 79 ± 6% [^99m^Tc]Tc-Z_HER3_-ABD-Z_HER3_-AA. Nonconjugated [^99m^Tc]Tc was removed by size-exclusion chromatography purification resulting in compounds with a radiochemical purity of >99% in both cases. A test to investigate the stability of the label was performed by incubation with a large excess of histidine, at room temperature or 37 °C. The results showed no significant release of [^99m^Tc]Tc during the 4 h observation period for [^99m^Tc]Tc-Z_HER3_-ABD-Z_HER3_-mcDM1 or [^99m^Tc]Tc-Z_HER3_-ABD-Z_HER3_-AA (Table S1).

### Cell Binding and Uptake Kinetics

**Figure 3:**
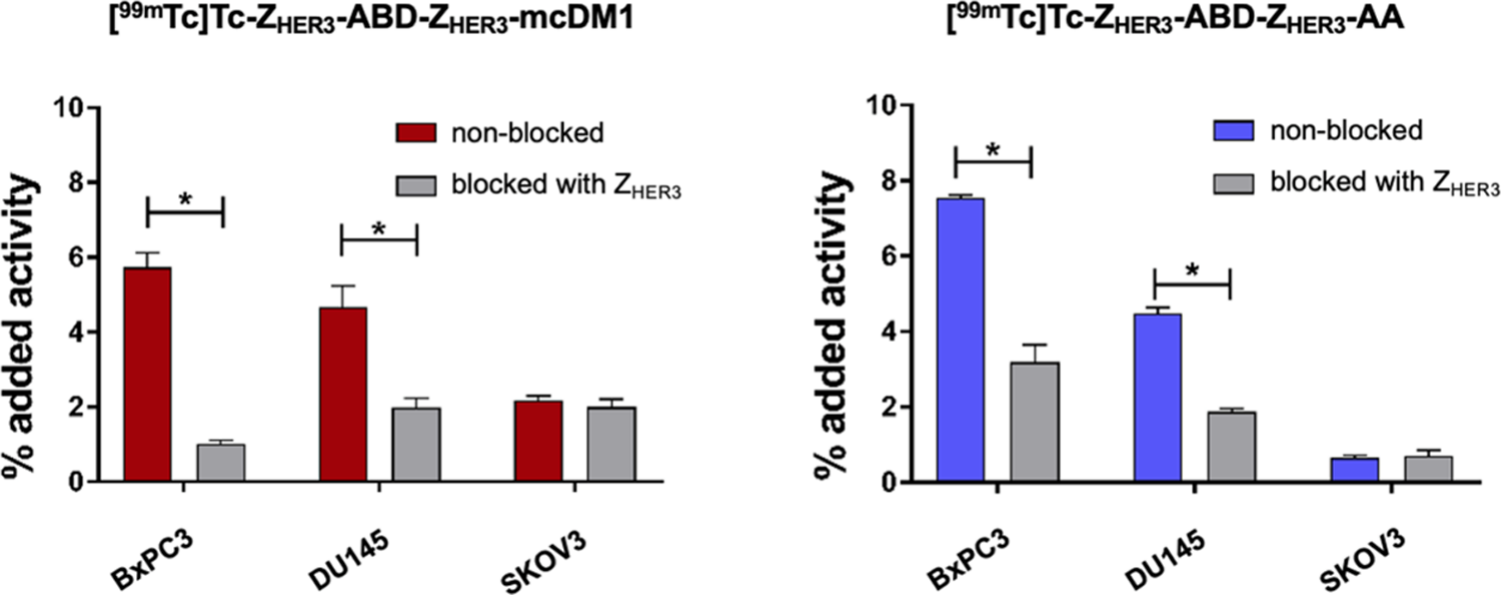
*In vitro* specificity. The HER3-expressing cell lines BxPC3 and DU145 and the HER3-very low SKOV3 cells were incubated with 0.1 nM of radiolabeled conjugates for 1 h at 37 °C. Cells in the blocked group were incubated with 100 nM of nonlabeled HER3-binding affibody prior to incubation with the radiolabeled conjugates. The data are displayed as the average ± SD (*n* = 3). *statistically significant difference (*p* < 0.05).

The radiolabeled constructs were further characterized by analysis of their ability to interact with the HER3-positive BxPC3, DU145, and HER3-very low SKOV3 cell lines. The cells were incubated with the constructs, with or without preblocking of available HER3 receptors with an excess of unlabeled Z_HER3_. From this analysis (Figure 3) it could be observed that the binding of [^99m^Tc]Tc-Z_HER3_-ABD-Z_HER3_-mcDM1 and [^99m^Tc]Tc-Z_HER3_-ABD-Z_HER3_-AA to the cells was significantly (*p* < 0.05) decreased after preblocking, compared with the nonblocked cells. For the HER3-very low SKOV3 cells, there was, as expected, no difference in uptake between the nonblocked and blocked cells. Additionally, the amount of associated radioactivity with the SKOV3 cells was significantly lower than the associated radioactivity with the BxPC3 and DU145 cells (nonblocked groups), further showing the HER3-dependent nature of the cell binding.

The kinetics of the interactions between the constructs and the HER3-expressing BxPC3 cell line were measured in real-time using a LigandTracer instrument. The results showed that both radiolabeled constructs had similar apparent association and dissociation rates and similar apparent affinity (*K*_D_ values) of around 300 pM (Table S2).

Furthermore, the association and internalization kinetics of Z_HER3_-ABD-Z_HER3_-mcDM1 and Z_HER3_-ABD-Z_HER3_-AA in BxPC3 and DU145 cells were studied over 24 h (Figure S3). The association pattern of the constructs was similar in both cell lines. After 6 h, more than 50% of the maximum cell-bound activity was associated with the cells. For both cell lines, the internalized fraction increased with time. After 24 h of incubation, the internalized fractions were 39 ± 6% of the maximum cell-bound activity and 19 ± 1% of the maximum cell-bound activity for BxPC3 and DU145, respectively. The results showed that the internalized fraction of [^99m^Tc]Tc-Z_HER3_-ABD-Z_HER3_-mcDM1 in BxPC3 cells at 24 h was significantly higher than the internalized fraction in DU145 cells.

### Biodistribution

**Figure 4:**
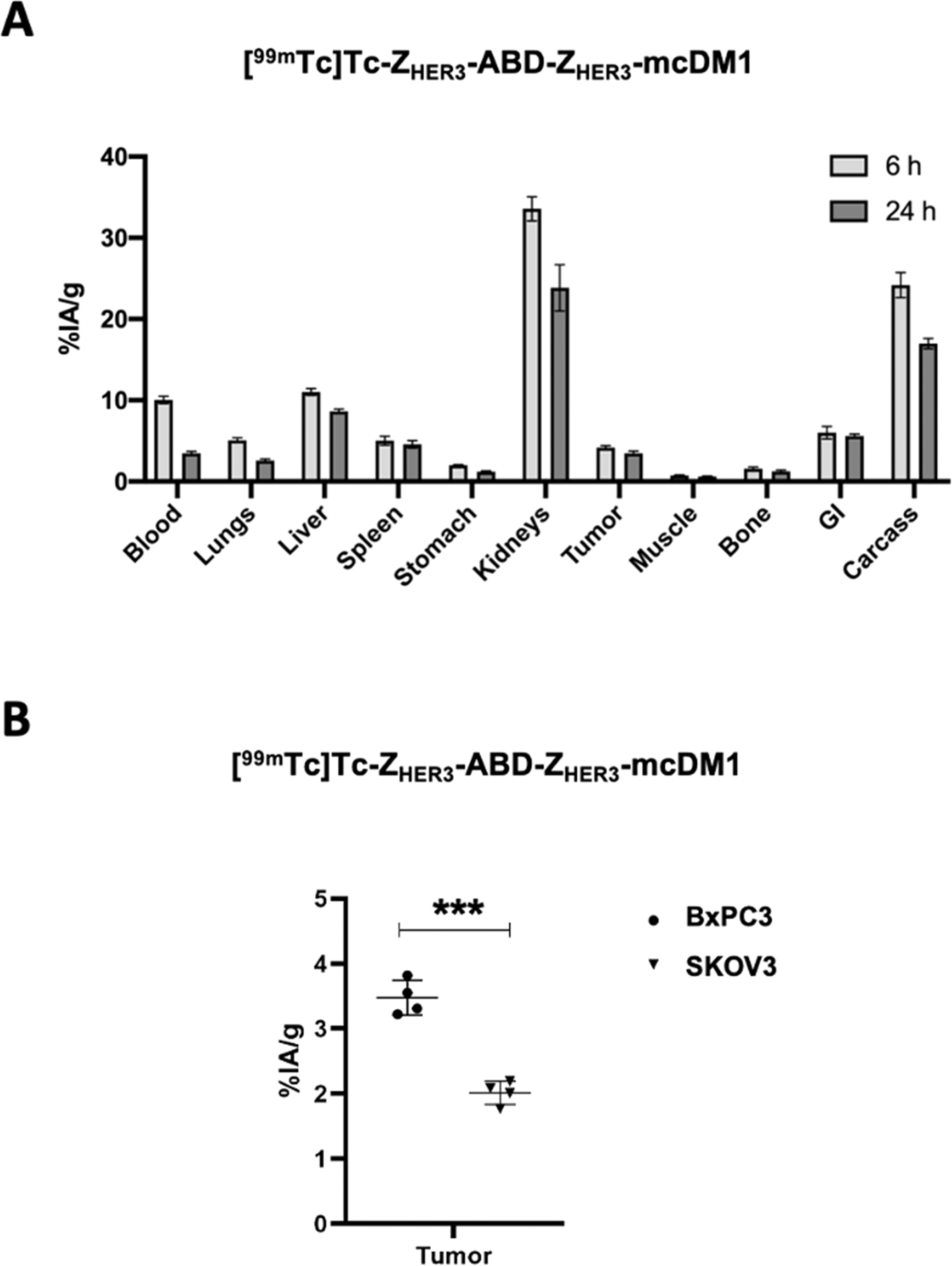
*In vivo* biodistribution in tumor-bearing mice. (A) The biodistribution of [^99m^Tc]Tc-Z_HER3_-ABD-Z_HER3_-mcDM1. The percent of injected activity per gram tissue (%IA/g) of various organs at 6 h pi is shown in light gray bars. The dark gray bars represent the %IA/g at 24 h pi. The GI and carcass values represent the %IA per the whole sample. (B) The uptake of [^99m^Tc]Tc-Z_HER3_-ABD-Z_HER3_-mcDM1 in HER3-positive BxPC3 tumors and the SKOV3 tumors. The values represent the %IA/g of the tumors at 24 h pi. *** denotes *p* < 0.001.

The drug conjugate, [^99m^Tc]Tc-Z_HER3_-ABD-Z_HER3_-mcDM1, was further analyzed *in vivo* in a mouse model with HER3-expressing BxPC3 tumors. The conjugate was injected, and its biodistribution was determined over time (Figure 4A). The results showed high retention in blood at 6 h pi that decreased at 24 h pi (10.1 ± 0.5 *vs* 3.5 ± 0.3% of injected activity per gram (%IA/g)). The uptake in kidneys at 6 h pi was 33.6 ± 1.5%IA/g, and the uptake in the liver was 11.0 ± 0.4%IA/g. The uptake in the BxPC3 tumors was moderate, with 4.2 ± 0.2%IA/g at 6 h pi, slightly decreasing over time to 3.5 ± 0.3%IA/g at 24 h pi. The BxPC3 tumor uptake at 24 h pi was significantly higher than the uptake in the SKOV3 (HER3-very low) tumors (3.5 ± 0.3 *vs* 2.0 ± 0.2%IA/g, *p* < 0.001), as shown in Figure 4B.

### Experimental *In Vivo* Therapy

To investigate the antitumor effect of Z_HER3_-ABD-Z_HER3_-mcDM1, mice bearing HER3-expressing BxPC3 tumors were treated with a combination of the previously evaluated cytostatic Z_HER3_-ABD-Z_HER3_ construct and Z_HER3_-ABD-Z_HER3_-mcDM1. The control groups were treated with monotherapy using Z_HER3_-ABD-Z_HER3_ or with the vehicle (PBS) 3 times per week. Taking into account a moderate uptake of Z_HER3_-ABD-Z_HER3_-mcDM1 in the liver and its potent cytotoxic effect in BxPC3 cells *in vitro*, the administration schedule in the combination treatment group was set to two injections of Z_HER3_-ABD-Z_HER3_ followed by one injection of Z_HER3_-ABD-Z_HER3_-mcDM1 per week to minimize potential off-tumor toxicities.

**Figure 5:**
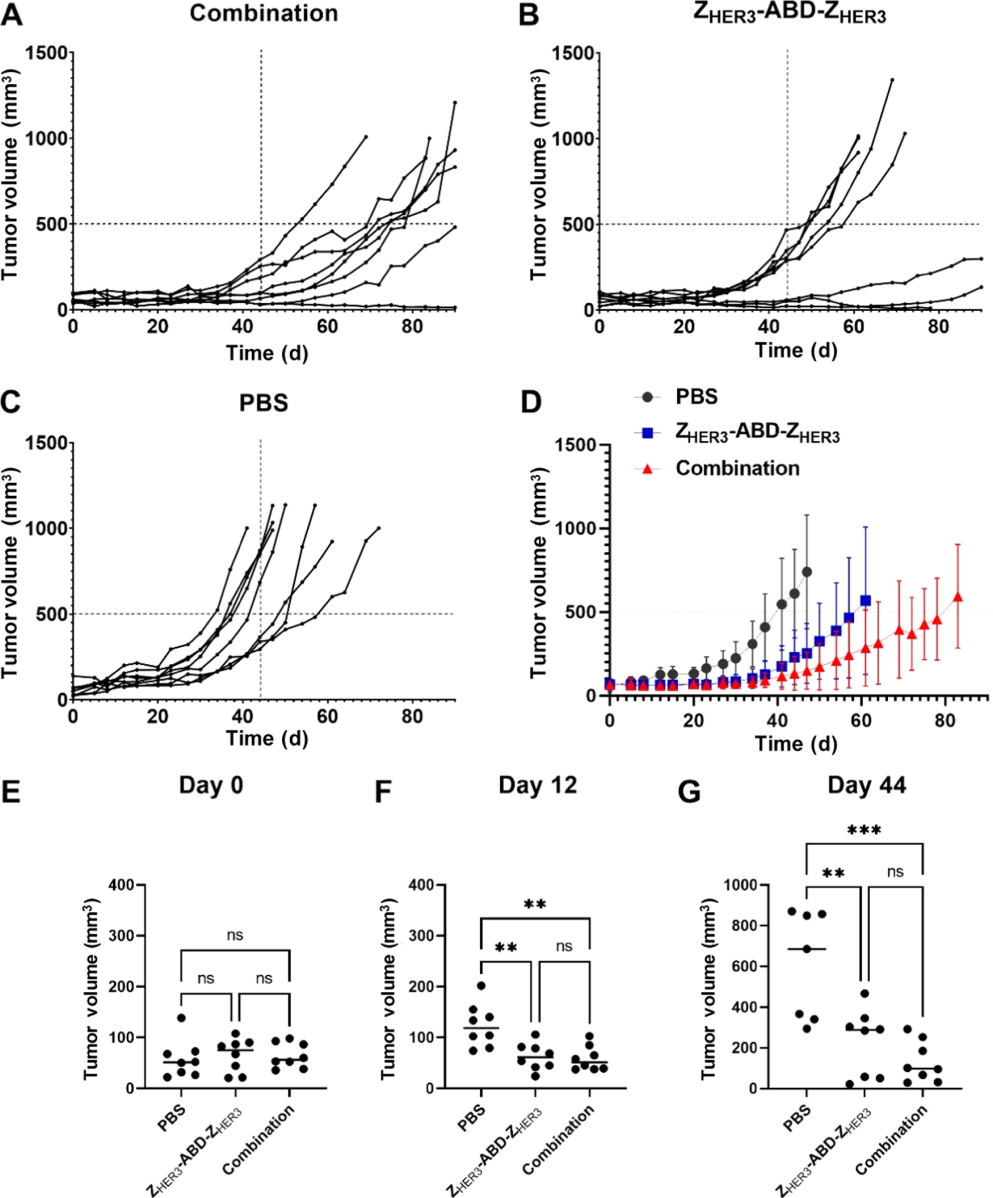
Tumor growth curves during experimental therapy in BALB/c nu/nu mice bearing BxPC3 xenografts, that received (A) a combination of Z_HER3_-ABD-Z_HER3_ and Z_HER3_-ABD-Z_HER3_-mcDM1, (B) only Z_HER3_-ABD-Z_HER3_, (C) PBS (vehicle control). The average tumor volume growth curves (D) were drawn until 38% of the mice in a group (3 out of 8) were euthanized. The mice were euthanized when the tumor volume exceeded the limit of 1000 mm^3^ or ulceration on the xenografts was observed. The vertical gridlines for A-D were set to day 44 when the first mouse in the control group was sacrificed, and the horizontal gridlines were set to a tumor volume of 500 mm^3^. Tumor volume comparison (E) at the start of therapy (day 0), (F) on day 12 and (G) on day 44; ** corresponds to *p* < 0.01, *** corresponds to *p* < 0.001.

The individual tumor growth curves are presented in Figure 5. The average tumor volumes at treatment start were 65 ± 25 mm^3^ (combination group), 65 ± 33 mm^3^ (monotherapy Z_HER3_-ABD-Z_HER3_), and 58 ± 38 mm^3^ (PBS), with no statistically significant differences between the tumor volumes. The tumor growth in the combination group (tumor doubling time 20 d, 95% CI from 16 to 23 d) was inhibited compared to the PBS group (tumor doubling time 11 d, 95% CI from 9 to 14 d) and the Z_HER3_-ABD-Z_HER3_ group (tumor doubling time 13 d, 95% CI from 10 to 16 d). The first significant differences between the tumor volumes were observed already on day 12, after two injections of Z_HER3_-ABD-Z_HER3_-mcDM1 and four injections of Z_HER3_-ABD-Z_HER3_ in the combination group and six injections of Z_HER3_-ABD-Z_HER3_ in the monotherapy group. The tumors in the combination group (59 ± 24 mm^3^) and Z_HER3_-ABD-Z_HER3_ group (63 ± 26 mm^3^) were significantly smaller (*p* < 0.01) than the tumors in the PBS group (124 ± 43 mm^3^). These differences were observed until day 47 when the first three mice were euthanized in the PBS group.

In the PBS group, all mice had exponential tumor growth and were euthanized due to the tumors reaching the limit of 1000 mm^3^. By day 72 all mice in this group had been euthanized. In the monotherapy group, five mice (62.5%) were euthanized by day 72 (four reached the tumor size limit and one had tumor ulceration), two mice (25%) had a growth delay with macroscopic tumors and one mouse (12.5%) had no visible tumor at day 90, when the study ended. In the combination group, four mice (50%) were euthanized by the end of the study on day 90 (three reached the tumor size limit and one had tumor ulceration), three mice (37.5%) had a growth delay with macroscopic tumors and one mouse (12.5%) had no visible tumor.

**Figure 6:**
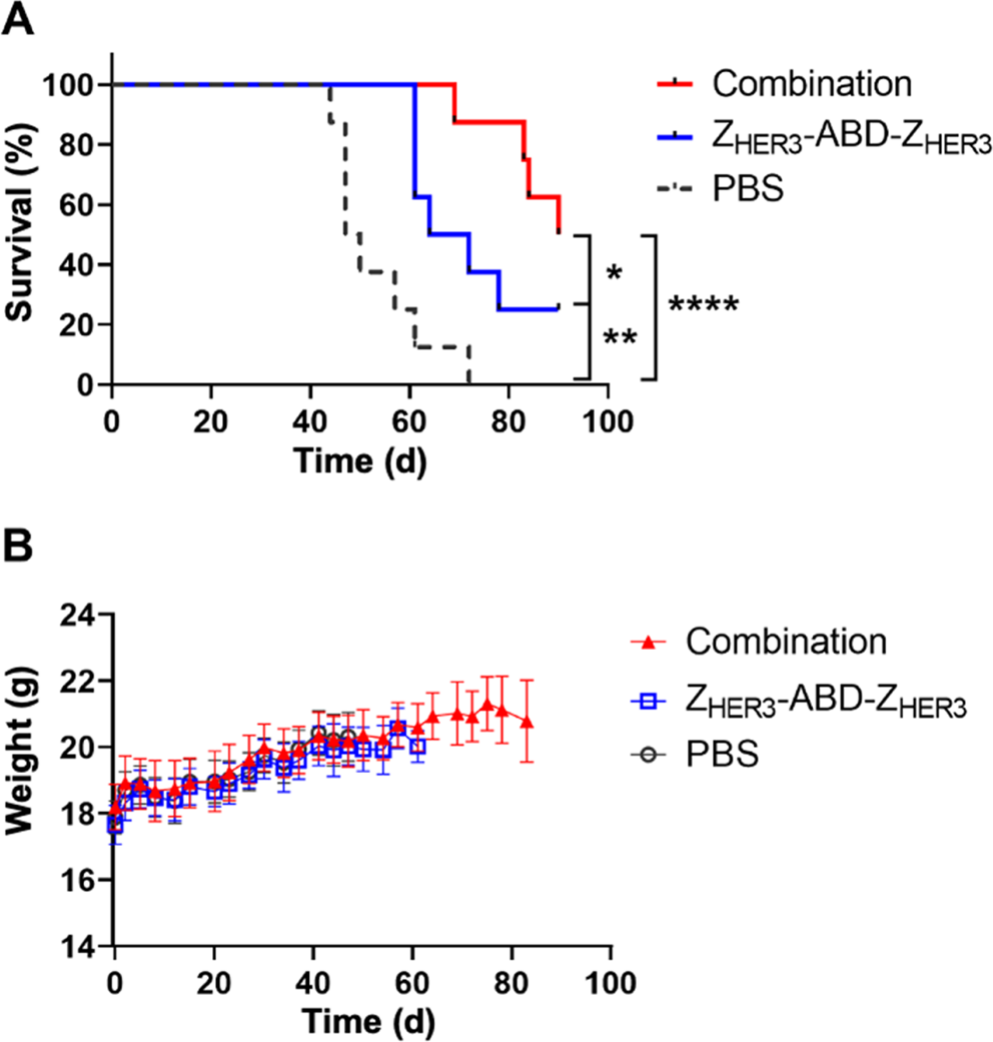
(A) Survival of BALB/c nu/nu mice (*n* = 8) bearing BxPC3 xenografts during experimental therapy treated with the combination of Z_HER3_-ABD-Z_HER3_ and Z_HER3_-ABD-Z_HER3_-mcDM1 (median survival 90 d, significantly longer than in monotherapy and PBS groups), Z_HER3_-ABD-Z_HER3_ (median survival 68 d, significantly longer than PBS group) and PBS (median survival 49 d). (B) The average animal weight in each group during therapy was presented as an average value ± SD.

The median survival in the combination group (90 d) was significantly longer than the median survival in the monotherapy group (68 d, *p* < 0.05) and in the PBS group (49 d, *p* < 0.001) (Figure 6A).

Both treatment strategies, the combination and monotherapy, were well-tolerated by the mice, without any observable side effects. No weight loss was recorded in any of the mice throughout the study (Figure 6B). Histopathological examination of the liver and kidneys from the mice in all groups concluded no toxic hepatic and renal treatment effects (Figure S4).

## Discussion

The human epidermal growth factor receptor 3 (HER3) is overexpressed at a relatively low level (up to 50.000 receptors/cell) compared to many other receptors targeted with drug conjugates and, therefore, poses a challenge for the design of efficient, targeted cytotoxic drugs. Nevertheless, its overexpression is associated with resistance to therapy and a poor prognosis for patients.^29^ Thus, HER3-targeted therapies could prove valuable for patients with tumors overexpressing the receptor. Efforts to create HER3-targeted therapies based on monoclonal antibodies and antibody-drug conjugates have undergone clinical evaluation but no drugs have yet been approved for clinical use. In light of the current unmet clinical need, we have developed affibody-based drug candidates targeting HER3.^24,27^ The aim of this study was to characterize a bivalent affibody drug conjugate, Z_HER3_-ABD-Z_HER3_-mcDM1.

The fusion protein, Z_HER3_-ABD-Z_HER3_-E_3_C, could be readily expressed recombinantly, and purified to homogeneity, followed by efficient mcDM1 conjugation. The final purification of Z_HER3_-ABD-Z_HER3_-mcDM1 by reversed-phase high-performance liquid chromatography yielded a monodisperse product of very high purity.

The determination of the kinetic constants of the interaction between the constructs, Z_HER3_-ABD-Z_HER3_-mcDM1, Z_HER3_-ABD-Z_HER3_-AA, and Z_HER3_-ABD-mcDM1, and HER3, revealed strong interactions, with equilibrium dissociation constants (*K*_D_ values) ranging from 3 to 4 nM. It was considerably weaker than the interaction between Z_HER3_ (without fusion partner) and HER3, which has earlier been determined to 21 pM,^21^ but was still considered strong enough to allow *in vivo* targeting of HER3-expressing tumors. An earlier study has also shown a weaker affinity of Z_HER3_ for HER3 when it was part of a fusion protein.^23^ In that study, Z_HER3_-ABD-Z_HER3_ had a *K*_D_ value of 1.1 nM, which was similar to the affinity determined for Z_HER3_-ABD-Z_HER3_-mcDM1 in this study (*K*_D_ 3.2 nM). The result shows that the addition of mcDM1 has only a minor effect on the affinity for HER3. The affinity for mouse ErbB3 was four to 10-fold weaker than for HER3 and ranged from 12 to 38 nM. The experiment was conducted with immobilized drug conjugate on the sensor chip followed by injection of HER3 or mErbB3 to be able to employ a 1:1 interaction model. This setup does not reveal the possible avidity effect of using two affibody molecules in Z_HER3_-ABD-Z_HER3_ and Z_HER3_-ABD-Z_HER3_-mcDM1.

Even though the interaction with mErbB3 was weaker, it would likely be strong enough to indicate the on-target uptake in different normal organs when investigating the biodistribution in mice. However, the uptake in normal organs in humans could be expected to be higher if endogenous HER3 expression is similar to the expression level of mErbB3 in mice.

The cytotoxic effect on the pancreatic cancer cell line BxPC3, with moderate HER3 expression, showed an IC_50_ value of 3 nM for Z_HER3_-ABD-Z_HER3_-mcDM1. It was more potent than Z_HER3_-ABD-mcDM1, which had an IC_50_ value of 14 nM in this study and 7 nM in an earlier study.^27^ An earlier study on Z_HER3_-ABD-Z_HER3_ showed an IC_50_ value of 0.5 nM on the same cell line.^26^ However, the effect of Z_HER3_-ABD-Z_HER3_ on the BxPC3 cells was cytostatic, whereas the effect of Z_HER3_-ABD-Z_HER3_-mcDM1 is cytotoxic. Thus, the IC_50_ values for cell viability may not be completely indicative of the biological effect on tumor growth *in vivo*. The DU145 cell line, with low HER3 expression, was less affected by Z_HER3_-ABD-Z_HER3_-mcDM1 and Z_HER3_-ABD-mcDM1 than the BxPC-3 cells, and a clear cytotoxic effect could only be observed at the highest concentration of 1500 nM. For the HER3-very low SKOV3 cell line, a cytotoxic effect could be seen at the highest concentration. Earlier studies on HER2-targeting drug conjugates based on affibody molecules, and including DM1, have shown that receptor-negative cell lines are sometimes affected by concentrations at 1000 nM and above, so the cytotoxic action by Z_HER3_-ABD-Z_HER3_-mcDM1 on SKOV3 in this study likely stems from unspecific uptake by the cells.

The drug conjugate Z_HER3_-ABD-Z_HER3_-mcDM1 was efficiently radiolabeled with technetium-99m to perform quantitative characterization *in vitro* and *in vivo*. [^99m^Tc]Tc-Z_HER3_-ABD-Z_HER3_-mcDM1 was found to bind to both BxPC3 and DU145 cells. The binding was blockable by presaturation of available HER3 receptors with nonradiolabeled Z_HER3_ confirming that the cell interaction was indeed HER3-mediated. The nonblocked binding to DU145 cells was slightly lower than to BxPC3 cells which indicates that the DU145 cells express HER3 receptors to a lower level than BxPC3. The binding to the HER3-very low SKOV3 cells was significantly lower, and the binding was not blockable. The activity uptake for SKOV3 cells might correspond to the background level in the experiment. Furthermore, [^99m^Tc]Tc-Z_HER3_-ABD-Z_HER3_-mcDM1 was internalized by both BxPC3 cells and DU145 cells, with a faster internalization rate for BxPC3 (Figure S3). It was, therefore, not surprising that the cytotoxic effect of Z_HER3_-ABD-Z_HER3_-mcDM1 on DU145 cells was significantly weaker than the cytotoxic effect on BxPC3 cells. Similar differences in the cytotoxic action of antibody-drug conjugates (ADCs) on different cell lines have been observed in other studies. For example, during the development of trastuzumab emtansine, the IC_50_ values differed by more than 10-fold on SKBR3 and BT474 cells, both with similar and high overexpression of HER2.^30^ HER2-targeted drug conjugates, based on affibody molecules, have also shown a similar behavior.^31^ For ADCs, the difference in cytotoxic action has been attributed to differences in the rate of receptor internalization, lysosomal transport, and degradation. Furthermore, cell lines may develop resistance by expression of, for example, multidrug resistance proteins.^32−34^ We observed some differences in HER3 expression and rate of internalization between BxPC3 and DU145 cells, but other factors such as those described above, also likely play a role.

The internalization rate of [^99m^Tc]Tc-Z_HER3_-ABD-Z_HER3_-mcDM1 by BxPC3 cells was similar to the previously studied divalent construct without drug, [^99m^Tc]Tc-Z_HER3_-ABD-Z_HER3_^23^ and the monovalent drug conjugate, [^99m^Tc]Tc-Z_HER3_-ABD-mcDM1.^27^ The internalized fraction was around 20% of the maximal cell-associated activity after 6 h of incubation. The level of internalized activity at this time point was similar to previously studied indium-111-labeled Z_HER3_-ABD-Z_HER3_ (*ca.* 20% at 8 h) and [^99m^Tc]Tc-Z_HER3_-ABD-mcDM1 (*ca.* 25% at 8 h).^27^ After 24 h, the internalized fraction of [^99m^Tc]Tc-Z_HER3_-ABD-Z_HER3_-mcDM1 was *ca.* 40%, which suggested efficient drug delivery. It was comparable, although, slightly lower than the level previously reported for [^111^In]In-Z_HER3_-ABD-Z_HER3_ (*ca.* 50% at 24 h).^23^

The apparent affinity of [^99m^Tc]Tc-Z_HER3_-ABD-Z_HER3_-mcDM1 and [^99m^Tc]Tc-Z_HER3_-ABD-Z_HER3_-AA to BxPC3 cells was determined to be 0.3 nM, which was 10-fold stronger than the affinity measured between the constructs and the extracellular domain of HER3 in the biosensor (Table 1). The affinity was similar to the previously determined affinity of [^99m^Tc]Tc-Z_HER3_-ABD-mcDM1 for BxPC3 cells (0.2 nM).^27^ The result shows that the higher cytotoxic potency of [^99m^Tc]Tc-Z_HER3_-ABD-Z_HER3_-mcDM1 compared to [^99m^Tc]Tc-Z_HER3_-ABD-mcDM1 was not a consequence of the affinity for the cells but a consequence of a step in the subsequent poisoning process. The affinity of indium-111-labeled Z_HER3_-ABD-Z_HER3_ for BxPC3 cells has earlier been determined to be 0.1 nM, which is not significantly different from the affinity of [^99m^Tc]Tc-Z_HER3_-ABD-Z_HER3_-mcDM1, which corroborates the biosensor result (Table 1) that addition of DM1 does not affect HER3 affinity significantly.

The biodistribution and tumor accumulation of [^99m^Tc]Tc-Z_HER3_-ABD-Z_HER3_-mcDM1 was similar to the biodistribution of the monovalent drug conjugate [^99m^Tc]Tc-Z_HER3_-ABD-DM1,^27^ however with some apparent differences. The activity accumulation of the monovalent variant at 24 h pi was roughly equal in the liver and the xenografts, while the activity accumulation of the bivalent variant was 2-fold higher in the liver than in xenografts. Thus, the bivalent conjugate demonstrated a higher degree of hepatobiliary excretion than the monovalent conjugate. This observation matches our prior observations of higher hepatic accumulation of multivalent anti-HER3 affibody constructs compared with monovalent constructs.^35^ Furthermore, the bivalent variant had a reduced accumulation in kidneys (*ca.* 20%IA/g at 24 h) compared to the monovalent variant (*ca.* 50%IA/g at 24 h^27^) and a similar accumulation as the nontoxic [^99m^Tc]Tc-Z_HER3_-ABD-Z_HER3_ (*ca.* 20%IA/g at 24 h).^23^ Comparison of the biodistribution of [^99m^Tc]Tc-Z_HER3_-ABD-Z_HER3_-mcDM1 and [^99m^Tc]Tc-Z_HER3_-ABD-Z_HER3,_^23^ showed that the drug conjugate has a higher liver uptake, which is likely a consequence of the hydrophobic nature of DM1. The liver accumulation of [^99m^Tc]Tc-Z_HER3_-ABD-Z_HER3_-mcDM1, may become a limiting factor if high and repeated doses are to be given. However, published data for anti-HER2 and anti-EpCAM drug conjugates based on ESPs (affibody molecules, ADAPTs, and DARPin), have also shown some accumulation in the liver and the kidneys. In those experimental therapy studies, significant therapeutic effects were recorded without any detectable liver or kidney toxicity, or any significant weight loss (affibody molecules);^14,31,36,37^ ADAPT;^15,38^ DARPin.^39,40^ In one of the studies on a HER2-targeting affibody drug conjugate, Z_HER2_-ABD-mcDM1, a linker with the amino acid sequence Glu-Glu-Glu between the ABD and mcDM1, was found to decrease the hydrophobic character of the drug conjugate, and the unspecific *in vivo* uptake in the liver.^37^ To minimize liver uptake, we used a Glu-Glu-Glu-linker between the ABD and mcDM1 in this study.

The promising data from the *in vitro* and *in vivo* evaluation motivated further investigation of the therapeutic effect of Z_HER3_-ABD-Z_HER3_-mcDM1 in a xenograft model in mice. Based on the *in vivo* therapy results from our previous studies, we hypothesized that the cytostatic action of the Z_HER3_-ABD-Z_HER3_ on BxPC3 xenografts could be potentiated by adding a cytotoxic component: Z_HER3_-ABD-Z_HER3_-mcDM1. Due to a moderate liver uptake of Z_HER3_-ABD-Z_HER3_-mcDM1 and its potent cytotoxicity in BxPC3 cells, the combination treatment included two injections of Z_HER3_-ABD-Z_HER3_ followed by one injection of Z_HER3_-ABD-Z_HER3_-mcDM1 every week to minimize off-tumor toxicities. The combination treatment significantly reduced the average tumor volume compared to the control group receiving only PBS injections, starting already from day 12. There was also a tendency, although not statistically significant, for smaller tumor volumes in the group receiving the combination treatment compared to the group receiving Z_HER3_-ABD-Z_HER3_ monotherapy. The combination treatment significantly increased the median survival of mice in comparison to both the monotherapy and the vehicle control groups.

The results from the preclinical therapy experiments are promising and of particular interest primarily for two reasons. First, even though the number of HER3 receptors on the cancer cells was relatively low in comparison to *e.g.*, HER2, it was sufficiently high to produce a pronounced antitumor effect *in vivo* for the combination group. Second, there was no observable toxicity (no weight loss, no histopathological changes in liver and kidneys) over the 90 days of continuous treatment using Z_HER3_-ABD-Z_HER3_-mcDM1, despite moderate uptake in normal organs such as the liver and kidneys. This indicates that monotherapy with Z_HER3_-ABD-Z_HER3_-mcDM1, using higher doses or a more frequent administration schedule, could potentially provide an even stronger antitumor effect and is of interest to investigate in future studies.

In conclusion, Z_HER3_-ABD-Z_HER3_-mcDM1 is a potent HER3-specific drug conjugate with a favorable biodistribution, showing additive effects to Z_HER3_-ABD-Z_HER3_ in an experimental therapy model of xenografted pancreatic BxPC3 cells in mice. It holds promise for further clinical evaluation.

## Experimental Section

### General

Unless otherwise noted, all chemicals were purchased from Sigma-Aldrich (St. Louis, MO) or Merck (Darmstadt, Germany). HER3 and murine ErbB3 (mErbB3) were purchased from Sino Biological (Wayne, PA). Restriction digestion enzymes were obtained from New England Biolabs (Ipswich, MA). All compounds are >95% pure by reversed-phase high-performance liquid chromatography (RP-HPLC) analysis.

### Design of Affibody Constructs

The HER3-binding affibody molecule Z_HER3:08698_^21^ was used in this study, henceforth referred to as Z_HER3_. The ABD used was ABD_035_ with subpicomolar affinity to human serum albumin.^20^ Genes encoding two fusion proteins, Z_HER3_-ABD-Z_HER3_-E_3_C, and Z_HER3_-ABD-E_3_C, were synthesized and inserted into the pET26b(+) plasmid vector. A DNA sequence encoding a tag with the peptide sequence Met-His-Glu-His-Glu-His-Glu, a (HE)_3_-tag, was placed in the 5′-end, and nucleotides encoding the amino acid sequence Gly-Gly-Gly-Ser was added between the affibody domain(s) and ABD. A DNA sequence encoding the amino acids Glu-Glu-Glu-Cys was placed in the 3′-end of both genes. The integrity of the gene constructs was confirmed by DNA sequencing.

### Expression and Purification of the Affibody Fusion Proteins

The fusion proteins were produced intracellularly in *E. coli*, strain BL21*(DE3) (New England Biolabs). The cells were grown in Tryptic Soy Broth (TSB, 30 g/L) containing yeast extract (5 g/L) and kanamycin (50 mg/L) at 37 °C. The production of the proteins was induced with isopropyl β-D-1-thiogalactopyranoside (IPTG; Appolo Scientific, Stockport, U.K.) with a final concentration of 1 mM. After induction, the cells were incubated at 25 °C for 16 h and harvested. The cells were lysed using a sonicator and purified by affinity chromatography on a HiTrap NHS Sepharose column with immobilized human serum albumin (HSA). The buffer for equilibration and washing contained 25 mM Tris-HCl, 1 mM EDTA, 200 mM NaCl, 0.05% Tween-20, pH 8.0 (TST buffer). The cell extract after sonication was cleared by centrifugation followed by filtration through a 0.45 μm filter (Pall, Port Washington, NY) and then loaded onto the HSA-column. The column was washed with 10 column volumes (CV) of TST buffer and 10 CV of ammonium acetate buffer (5 mM, pH 5.5), followed by elution with acetic acid (0.5 M, pH 2.8). Fractions containing eluted proteins were pooled and lyophilized, and stored at −20 °C.

### Conjugation with DM1

The lyophilized proteins were reconstituted in PBS buffer (pH 6.5) to a concentration of 100 μM, and potentially oxidized cysteine residues were reduced with 5 mM tris(2-carboxyethyl) phosphine (TCEP) for 30 min at 37 °C. Subsequently, mcDM1 (Levena Biopharma, San Diego, CA) was added to the proteins at a molar ratio of 2:1, followed by incubation overnight at room temperature, yielding Z_HER3_-ABD-Z_HER3_-mcDM1 and Z_HER3_-ABD-mcDM1.

A nontoxic control protein, Z_HER3_-ABD-Z_HER3_-AA, was generated by alkylation of the C-terminal cysteine of Z_HER3_-ABD-Z_HER3_-E_3_C with 2-iodoacetamide. The lyophilized protein was dissolved in 200 mM NH_4_HCO_3_ (pH 8.0) buffer. Potentially oxidized cysteine residues were reduced by the addition of TCEP to a final concentration of 10 mM with incubation at 55 °C for 1 h. After the incubation, 2-iodoacetamide was added to a final concentration of 20 mM, followed by incubation at room temperature for 30 min in the dark.

Z_HER3_-ABD-Z_HER3_-mcDM1, Z_HER3_-ABD-mcDM1, and Z_HER3_-ABD-Z_HER3_-AA were purified by reversed-phase high-performance liquid chromatography (RP-HPLC). Prior to loading on a Zorbax SB-C18 column (Agilent, Santa Clara, CA), the buffers of the protein solutions were changed to HPLC buffer A (0.1% trifluoroacetic acid in water). The constructs were loaded on the column with HPLC buffer A as the running buffer, followed by washing with HPLC buffer A and elution with a gradient from 20% to 60% HPLC buffer B (0.1% trifluoroacetic acid in acetonitrile) for 40 min. Fractions containing the constructs were collected, pooled, and lyophilized.

### Characterization of the Constructs

The lyophilized constructs after RP-HPLC purification were dissolved in PBS (pH 7.4), and the concentrations were determined by a BCA assay kit (Thermo Fisher Scientific, Waltham, MA). Subsequently, the constructs were analyzed by RP-HPLC using an analytical Zorbax 300SB-C18 column (Agilent Technologies), where the column was eluted with a 20–60% gradient of acetonitrile in water, supplemented with 0.1% trifluoroacetic acid over 40 min. Furthermore, the conjugates were separated with size-exclusion chromatography under native conditions using a Superdex 75 5/150 column (GE Healthcare, Uppsala, Sweden) with PBS as the running buffer to analyze the mono/oligomeric state.

### Surface Plasmon Resonance Binding Analysis

The affinity of the constructs to HER3 and mouse ErbB3 was investigated on a Biacore T200 system (Cytiva, Uppsala, Sweden) using a CM5 chip with immobilized Z_HER3_-ABD-Z_HER3_-mcDM1, Z_HER3_-ABD-mcDM1, and Z_HER3_-ABD-Z_HER3_-AA in different flow-cells. PBS with 0.05% Tween-20 (PBS-T) was used as a running buffer. The flow rate was 50 μL/min. The chip was regenerated after each injection with 20 mM HCl for 30 s. HER3 and mErbB3 were injected at concentrations ranging from 6 to 100 nM. The association and dissociation rates were derived using a Langmuir 1:1 kinetics model in the T200 evaluation software.

### Cell Culture

The human cancer cell lines of BxPC3 (pancreatic cancer), DU145 (prostate cancer), and SKOV3 (ovarian cancer) were grown in RPMI-1640 (BxPC3, DU145) or McCoy’s 5A (SKOV3) media, supplemented with 1% Penicillin/Streptomycin and 10% Fetal bovine serum in a 5% CO_2_ humidified incubator at 37 °C.

### *In Vitro* Cytotoxicity

The cytotoxicity of Z_HER3_-ABD-Z_HER3_-mcDM1, Z_HER3_-ABD-mcDM1, and Z_HER3_-ABD-Z_HER3_-AA on the BxPC3, DU145, and SKOV3 cell lines was investigated by incubating the cells with dilution series of the constructs. The cells were seeded at 5000 cells (BxPC3 and DU145) or 2000 cells (SKOV3) per well in a 96-well plate and allowed to attach overnight. The following day, the medium was changed to a medium containing different concentrations of the constructs, followed by incubation for 3 days at 37 °C. The viability of the cells was measured using a Cell Counting Kit-8 (Sigma-Aldrich).

### Labeling with [^99m^Tc]Tc and Determination of the *In Vitro* Stability of the Label

[^99m^Tc]Tc, in the form of [^99m^Tc]TcO_4_^–^, was obtained through elution of an Ultra-TechneKow generator (Mallinckrodt, The Netherlands) with sterile 0.9% sodium chloride.

For labeling of Z_HER3_-ABD-Z_HER3_-mcDM1 and Z_HER3_-ABD-Z_HER3_-AA with [^99m^Tc]Tc on the N-terminal (HE)_3_-tag, [^99m^Tc]TcO_4_^–^ was first converted to tricarbonyl technetium-99m ([^99m^Tc]Tc(CO)_3_) using a CRS-kit (PSI, Villigen, Switzerland) according to a previously published protocol.^27^ Z_HER3_-ABD-Z_HER3_-mcDM1 (52.5 μg in 125 μL PBS) and Z_HER3_-ABD-Z_HER3_-AA (52.5 μg in 40 μL PBS) were incubated with 35–40 μL of [^99m^Tc]Tc(CO)_3_ (160–490 MBq) for 60 min at 50 °C. The radiochemical yield was determined by instant thin-layered chromatography (ITLC) with PBS elution. The ITLC strips were analyzed using a Cyclone Storage Phosphor Imager system (PerkinElmer, Waltham, MA). To analyze the presence of reduced hydrolyzed [^99m^Tc]Tc colloids, additional samples were eluted in pyridine:acetic acid:water (5:3:1.5). The radiolabeled conjugates were purified using Illustra NAP5 size-exclusion columns (Cytiva) pre-equilibrated with 1% BSA in PBS. After purification, the radiochemical purity was determined by ITLC.

To test the stability of the [^99m^Tc]Tc-label,^41^ 2 μg of the radiolabeled and purified conjugates were incubated in 50 μL PBS, with or without a 500-fold molar excess of histidine, at room temperature or 37 °C. After 1 and 4 h, samples were analyzed with ITLC to determine the percent of protein-associated activity.

### *In Vitro* Specificity

The *in vitro* binding specificity of [^99m^Tc]Tc-Z_HER3_-ABD-Z_HER3_-mcDM1 and [^99m^Tc]Tc-Z_HER3_-ABD-Z_HER3_-AA to the HER3-expressing BxPC3 and DU145 cells was determined. The very low-HER3-expressing SKOV3 cell line^42^ was included as a negative control. Cells were seeded in 3.5 cm Petri dishes at a density of 10^6^ cells/dish 1 day before the experiment. The cells were incubated with 0.1 nM of radiolabeled constructs for 1 h at 37 °C. Before the addition of radiolabeled constructs, half of the dishes were incubated with 100 nM of the monomeric HER3-targeting affibody molecule Z_HER3_ to block HER3 receptors on the cells. After incubation, the radioactive medium was removed, and the cells were collected. The radioactivity of the cell samples was measured in an automatic γ counter (Wallac 2480 Wizard; Wallac Oy, Turku, Finland). Each experiment was performed in triplicates.

### Measurement of Cell-Binding Kinetics with a LigandTracer Instrument

The apparent association rate (*k*_a_), dissociation rate (*k*_d_), and equilibrium dissociation constant (*K*_D_) were measured on HER3-expressing BxPC3 cells in real-time using a LigandTracer Yellow instrument (Ridgeview Instruments, Uppsala, Sweden). Cells were seeded in a dedicated area of a 10 cm Petri dish (approximately 3 × 10^6^ cells) one or 2 days before the experiment. The dish was mounted on the inclined, rotating holder of the LigandTracer instrument, and the baseline was recorded for 5–10 min. Afterward, either [^99m^Tc]Tc-Z_HER3_-ABD-Z_HER3_-mcDM1 or [^99m^Tc]Tc-Z_HER3_-ABD-Z_HER3_-AA were added in stepwise increasing concentrations (0.3, 1, 3 nM) to the culture medium. The next higher concentration was added when the binding had reached equilibrium at the current concentration. Subsequently, the solution with 3 nM construct was replaced with culture media, and the dissociation phase was recorded overnight. The data were analyzed with Tracedrawer software (Ridgeview Instruments) using a 1:1 binding model. The experiments were done in duplicate.

### Internalization of [^99m^Tc]Tc-Z_HER3_-ABD-Z_HER3_-mcDM1

To study the internalization of [^99m^Tc]Tc-Z_HER3_-ABD-Z_HER3_-mcDM1, BxPC3 and DU145 cells were continuously incubated with 0.1 nM of the construct at 37 °C. Samples were collected at 1, 2, 4, 6, and 24 h, and the membrane-bound and internalized fractions were collected using the “acid wash” method.^43^ In short, the cells were treated with 0.2 M glycine buffer (0.15 M NaCl, 4 M Urea, pH 2) for 5 min on ice. The solution was, after that, collected and the activity contained in this fraction was considered the membrane-bound fraction. The remaining activity was considered internalized and was collected after 30 min incubation with 1 M NaOH at 37 °C. All fractions were measured for activity content in an automatic γ counter. Each data point represents measurements from three individual experiments.

### Biodistribution

The animal studies were planned and performed according to the Swedish national legislation on the protection of laboratory animals. The experiments were approved by the local ethical committee for animal research in Uppsala, Sweden (permit 5.8.18–11931/2020, approved 28 August 2020). Twelve BALB/c nu/nu mice were implanted 3 weeks before the biodistribution study with 8 × 10^6^ BxPC3 cells (*n* = 8) or 8 × 10^6^ SKOV3 cells (*n* = 4, negative control). On the day of the experiment, the mice were injected with 41.5 μg of [^99m^Tc]Tc-Z_HER3_-ABD-Z_HER3_-mcDM1 (60 kBq, for the 6 h postinjection (pi) group and 450 kBq for the 24 h pi group). At the respective time point, the mice were euthanized, and the organs of interest were collected, weighed, and measured for their radioactivity content using an automatic γ counter.

### Experimental *In Vivo* Therapy

For the *in vivo* therapy experiment, BALB/c nu/nu mice were implanted with 5 × 10^6^ BxPC3 cells. The experimental therapy started 1 week after implantation when the xenografts had reached a measurable size. Two experimental groups (*n* = 8) were treated either exclusively with 77 μg (3.85 mg/kg) of Z_HER3_-ABD-Z_HER3_^26^ 3 times/week or with 77 μg of Z_HER3_-ABD-Z_HER3_ 2 times/week and 80 μg (4 mg/kg) (equimolar amount) of Z_HER3_-ABD-Z_HER3_-mcDM1, once per week. Both constructs were in PBS buffer. A control group (*n* = 8) was injected with PBS 3 times/week. The mice were monitored 2 times/week for weight, overall exterior, and tumor size. The tumor volumes were calculated by the formula 0.5 × *M*_long_ × (*M*_short_)^2^. Mice were excluded from the experiment if: (i) they lost >10% weight during 1 week or >15% of their total weight, (ii) the tumor ulcerated, (iii) the tumor volume exceeded 1 cm^3^. The treatment continued for 13 weeks, and on day 90, all surviving mice were sacrificed. The kidneys and livers of three animals from each group were collected and histologically evaluated.

### Statistical Analysis

Prism, version 8.2.1 (GraphPad Software, La Jolla, CA) was used for statistical analysis except to generate plots and perform statistical analysis of data from the *in vivo* therapy study when version 9.4.1 was used. Two values were compared using a Student’s *t* test, and multiple values were compared using a one-way analysis of variance (ANOVA) test with Bonferroni correction. Differences were considered significant when *p* < 0.05, unless other *p*-values are noted. A comparison of the survival curves in the experimental therapy study was performed using the Gehan-Breslow-Wilcoxon test giving more weight to events at early time points.

## Data Availability

Data will be made available on request.

## Supporting Information Available

Mass spectrometry; interaction; radiolabel stability; and internalization assays; as well as representative histopathological images (PDF)
