# Supplementary material for: Affibody-Drug Conjugates Targeting the Human Epidermal Growth Factor Receptor-3 Demonstrate Therapeutic Efficacy in Mice Bearing Low Expressing Xenografts
Source: ACS Pharmacol Transl Sci. 2024 Sep 12;7(10):3228–40. doi: 10.1021/acsptsci.4c00402 (PMC11475273; doi:10.1021/acsptsci.4c00402)
Supplement: Supplementary file 1 — pt4c00402_si_001.pdf [file pt4c00402_si_001.pdf]

Supporting Information

Affibody-Drug Conjugates Targeting the Human Epidermal Growth Factor Receptor-3 Demonstrate Therapeutic Efficacy in Mice Bearing Low Expressing Xenografts

Jie Zhang[1,§], Sara S. Rinne[2,§], Wen Yin[1], Charles Dahlsson Leitao[1], Elvira Björklund[2], Ayman Abouzayed[2], Stefan Ståhl[1], John Löfblom[1], Anna Orlova[2,3], Torbjörn Gräslund[1]*, Anzhelika Vorobyeva[4]*

[1]Department of Protein Science, KTH Royal Institute of Technology, Roslagstullsbacken 21, 114 17 Stockholm, Sweden

[2]Department of Medicinal Chemistry, Uppsala University, Dag Hammarskjöldsv 14C, 751 83 Uppsala, Sweden

[3]Science for Life Laboratory, Dag Hammarskjöldsv 14C, 751 83 Uppsala, Sweden

[4]Department of Immunology, Genetics and Pathology, Uppsala University, Dag Hammarskjölds väg 20, 751 85 Uppsala, Sweden

[§]These authors contributed equally to this work

*Torbjörn Gräslund, torbjorn@kth.se; Anzhelika Vorobyeva, anzhelika.vorobyeva@igp.uu.se

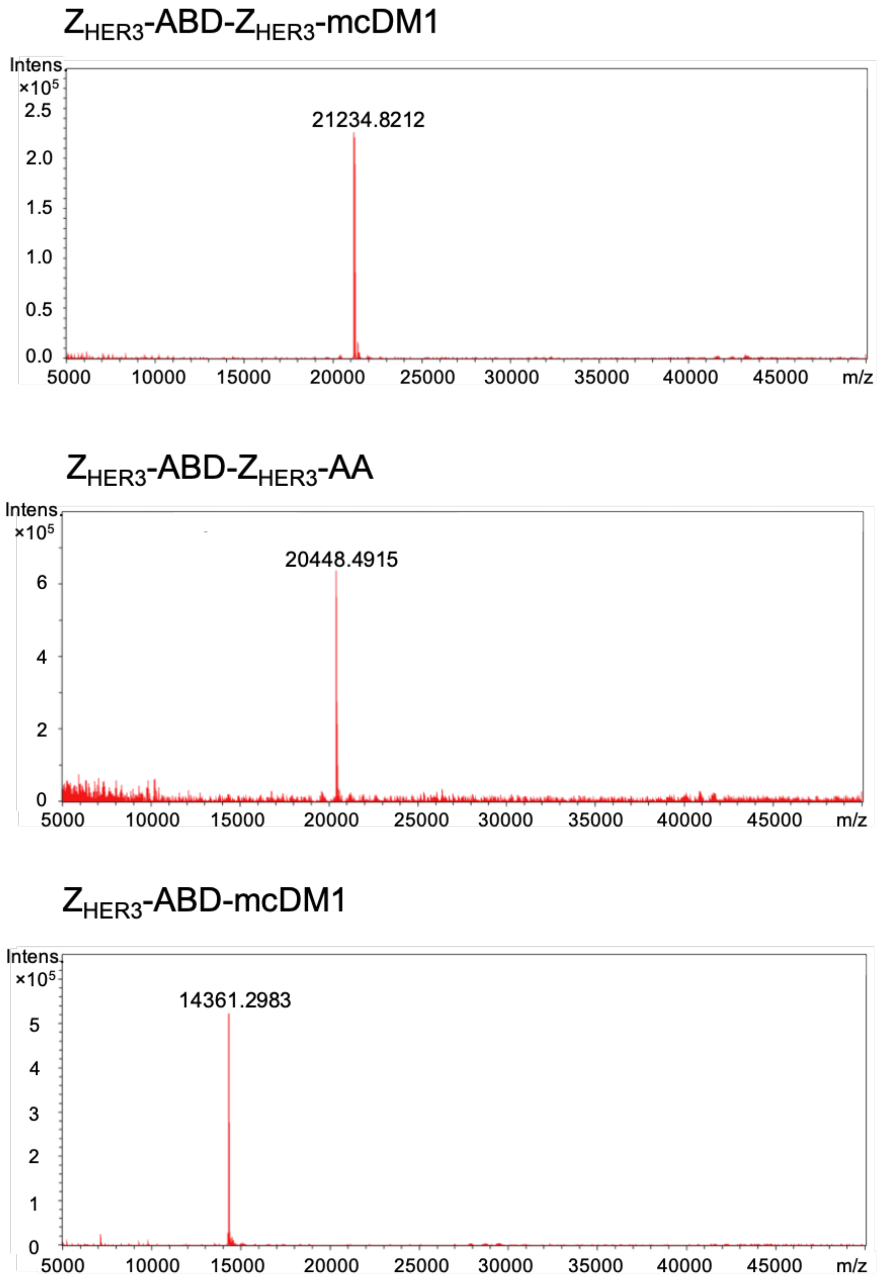

Supplementary Figure S1. LC-MS was used to determine the molecular masses (Da) of the constructs. Numbers in the panels indicate the m/z ratio, which is equal to the molecular weight assuming single charged species (z=1). The theoretical molecular weights are: 21234.30 g/mol (Z_{HER3}-ABD-Z_{HER3}-mcDM1), 20448.00 g/mol (Z_{HER3}-ABD-Z_{HER3}-AA), 14361.64 g/mol (Z_{HER3}-ABD-mcDM1).

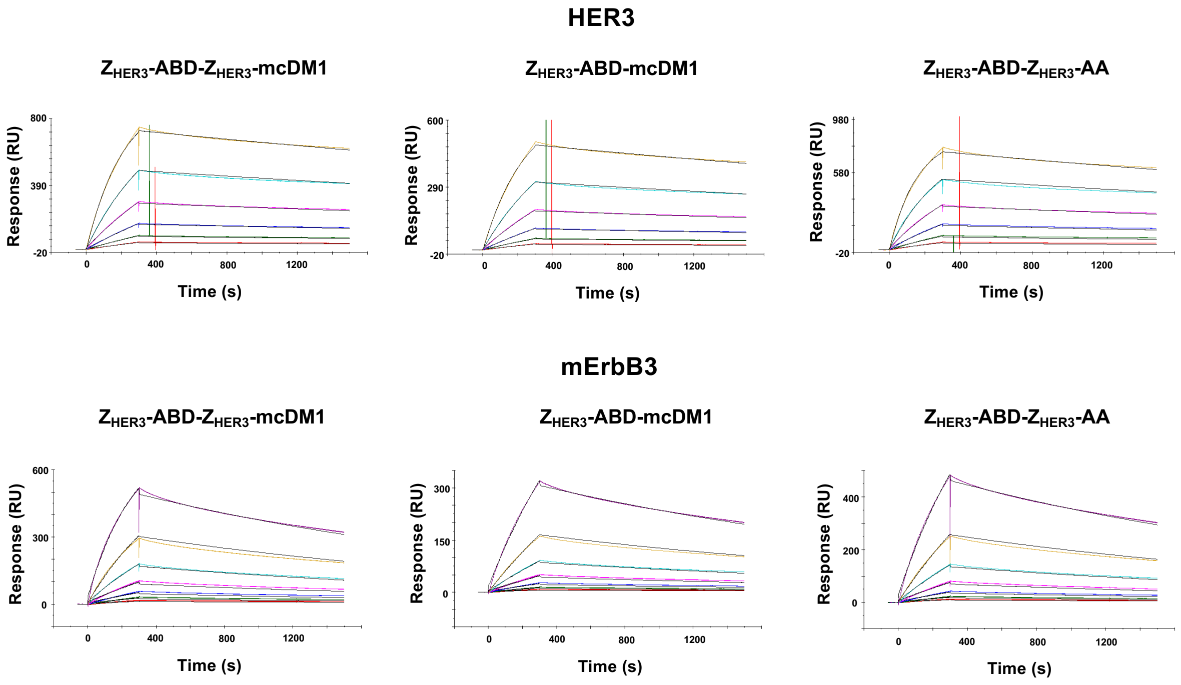

Supplementary Figure S2. SPR binding analysis. Two-fold dilution series of HER3 and mErbB3 (100 to 3 nM) were injected in serial-mode over flow-cells with immobilized Z_{HER3}-ABD-Z_{HER3}-mcDM1, Z_{HER3}-ABD-mcDM1, or Z_{HER3}-ABD-Z_{HER3}-AA. Each injection was done in duplicates. The panel shows an overlay of the sensorgrams (in color) obtained for each interaction. In black are the theoretical fitting curves.

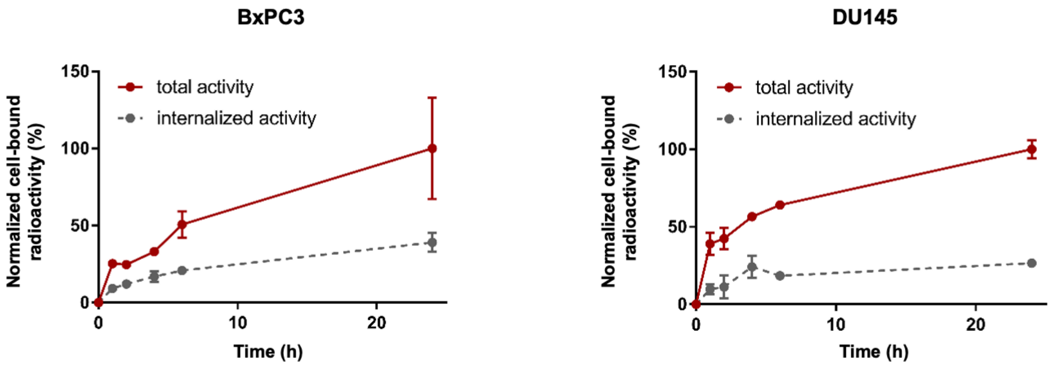

Supplementary Figure S3. Uptake and internalization of [^{99m}Tc]Tc-Z$_{HER3}$-ABD-Z$_{HER3}$-mcDM1 in BxPC3 and DU145 cells. Cells were continuously incubated with 0.1 nM of [^{99m}Tc]Tc-Z$_{HER3}$-ABD-Z$_{HER3}$-mcDM1. At different time points, the membrane-bound and internalized fractions were separated. Data are shown as the average ± SD (n=3).

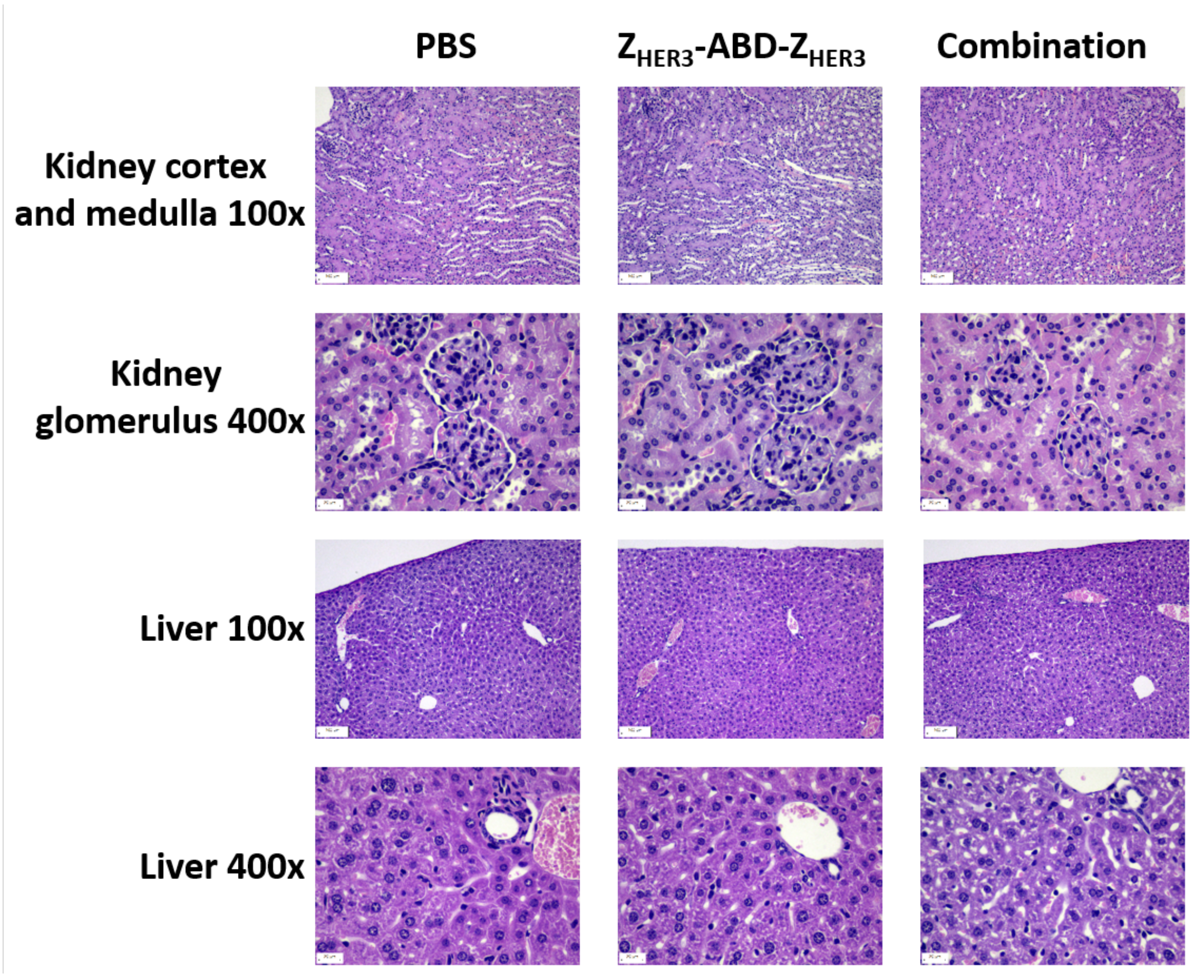

Supplementary Figure S4. Representative images of histopathological examination of kidneys and liver from the experimental in vivo therapy. H&E stain, 100x and 400x magnification of the kidneys and liver in mice from the PBS (vehicle) group, Z_{HER3}-ABD-Z_{HER3}-treated group, and the combination group (treated with Z_{HER3}-ABD-Z_{HER3} and Z_{HER3}-ABD-Z_{HER3}-mcDM1).

Supplementary Table S1. *In vitro* stability. Presented as % protein-associated activity (average ± SD, n=3 samples per data point).

Condition	[^{99m}Tc]Tc-Z_{HER3}-ABD-Z_{HER3}-mcDM1		[^{99m}Tc]Tc-Z_{HER3}-ABD-Z_{HER3}-AA	
	1 h	4 h	1 h	4 h
PBS, RT	99.9 ± 0.2	99.9 ± 0.1	99.9 ± 0.2	99.9 ± 0.1
Histidine, RT	99.7 ± 0.3	99.2 ± 0.7	99.7 ± 0.3	99.2 ± 0.7
Histidine, 37°C	99.4 ±0.2	98.2 ± 0.1	99.4 ± 0.2	98.2 ± 0.1

PBS = PBS buffer only, no free histidine present

RT = room temperature

Supplementary Table S2. Binding kinetics of [^{99m}Tc]Tc-Z_{HER3}-ABD-Z_{HER3}-mcDM1 and [^{99m}Tc]Tc-Z_{HER3}-ABD-Z_{HER3}-AA. The measurements were done on living BxPC3 cells in real-time using a LigandTracer instrument. The measurements were done in duplicates, and data was analyzed using TraceDrawer software using a 1:1 interaction model. The values are given as the average ± SD.

**Drug conjugates**	**k_a (1/Ms)**	**k_d (1/s)**	**K_D (pM)**
[^{99m}Tc]Tc-Z_{HER3}-ABD-Z_{HER3}-mcDM1	$1.3 \pm 0.1 \times 10^5$	$3.92 \pm 0.04 \times 10^{-5}$	314 ± 33
[^{99m}Tc]Tc-Z_{HER3}-ABD-Z_{HER3}-AA	$1.19 \pm 0.02 \times 10^5$	$3.8 \pm 0.3 \times 10^{-5}$	325 ± 28
